# Dr. M. L. Knapp on Cholera

**Published:** 1855-09

**Authors:** 


### DR. M. L. KNAPP ON CHOLERA.

Dr. M. L. Knapp has republished his essay, entitled "Discovery of the Cause, Nature, Cure, and Prevention of Cholera," which originally appeared in the New York Journal of Medicine, and has appended to it a considerable amount of new matter, some of which is in the shape of certificates testifying to the great merits of his paper. Altogether it forms a singular production. It purports to give the "*discovery*," not only of the cause, but of the cure and *prevention* of cholera. "If I have not explained everything pertaining to the subject," says the author, "I have given the *key* that *will explain everything;*" and he adds, "it is a consoling reflection that nations, cities and families can hereafter enjoy protection or immunity from the scourge, by simply conforming to the natural laws in regard to diet." And yet, in another place, he speaks of his "discovery" as a "*theory.*" "Until, then, *my theory* is disproved, and shown to be fallacious by the substitution of a more rational one (a moral impossibility I trow), it must stand as the true explanation of Cholera, to the great joy of the profession, and all mankind!!" The notes of admiration are ours. We do not wonder that a writer who expresses himself after this fashion should seek to fortify himself by such certificates as are given on the cover his pamphlet, in one of which, by Dr. W. Byrd Powell, Ex-Professor of the Memphis Eclectic Medical School, assures him that he is "the first who has shed light on this, hitherto, mysterious subject;" and in another, the author, Anson Jones, M. D., "Ex-President of the Republic of Texas,"

after relating that he has been with cholera "in Philadelphia, with it in New York, with it in New Orleans, and has seen much of it in Texas," that he has "*traveled* and *camped* with it," adds, that he "was *always successful* in the treatment of the disease." The Ex-President avers that he says this "without the least vanity, professional or otherwise," and his truly marvelous success he ascribes to the fact that he "had some faint glimmerings of the truth which you (Dr. Knapp) have made clear as the sun at noonday."

Can Dr. Knapp hope to gain anything with the profession by certificates such as these, or are they meant for another class of readers? Does he expect any medical man to believe that any one—ex-president, or ex-king—has been always successful in the treatment of cholera? But not to dwell upon the endorsement of Dr. Knapp's "discovery" or "theory."

The theory assumes that cholera is scurvy. For proof of the theory he cites facts and quotations, to his mind so conclusive, that he is sure those who cannot see therefrom "the identity of cholera and scurvy, would not be likely to acknowledge it though their patients were to rise from the dead and assert it." Dr. K. must pardon us—we confess ourselves of the number who discredit his "discovery," despite his array of facts and quotations, and in extenuation of our incredulity we would plead the following facts:

1st. Cholera has once and again visited many portions of our country where the diet of the people is as anti-scorbutic as Dr. Knapp could possibly desire or devise. It has prevailed in the best districts of Kentucky, among a rural population as well fed as any to be found on the face of the earth.

2d. Scurvy is an affection almost unknown in the interior of our country. It is of so rare occurrence that when cases have been met with we have known well-informed physicians to mistake them for other diseases. But during the spring of the present year scurvy was prevalent in Louisville. The drought of the previous year had cut off vegetation; the potato crop, turnips, cabbages, and all such esculents had failed. A number of persons died of scurvy before the nature of the disease was suspected by the physicians in attendance. Many cases were treated at the

Marine Hospital, and many were met with in private practice. But with them no cases of cholera occurred. Warm weather came while scurvy was yet prevailing, but still there was no cholera. We have had drought, failure of crops, scarcity of provisions, almost total lack of succulent vegetables, and, concurrently with this, scurvy in abundance, but no cholera. The causes which have always heretofore engendered the scorbutic diathesis, have here developed scurvy among the poorer class of our population, but they have utterly failed to originate cholera.

We conceive that one instance like this is fatal to Dr. Knapp's theory. Indeed, it is to us amazing that a man of sound mind can so abandon himself to such a fancy; or rather we should say it would be amazing if instances of the kind did not abound in the history of medicine. We do not wish to treat the labors of Dr. K. with disrespect; on the contrary we would encourage inquiries on the momentous subject of which the pamphlet before us treats, but when an author assumes a style and manner that brings to memory the boasted discoveries of Paracelsus or Basil Valentine, when he parades his guesses before the world as demonstrated truths in medicine, "the great joy of the profession and all mankind," and backs his pretentions by the certificates of post-masters, and ex-presidents, and professors and ex-professors in Eclectic Medical Schools, we must say that he is putting human patience to a severe test.

We hope Dr. Knapp had no thought of making merchandise of his discovery by sending it abroad in its present dress, but it cannot be denied that it comes in a most "questionable shape." Certainly he could not have intended the following sentence for medical readers: "Sanitary regulations can now be instituted that shall meet its (cholera's) invasions at the very threshold—in ship, camp, or city, and the public mind be so indoctrinated by suitable publications on the subject, that every family may know in what constitutes its safety; in fine, the true philosophy so disseminated, that the way-faring man, ihough a fool, need not err therein."

